# Nurturing Connection: The Emotional and Existential Dimensions of the Relationship Between Older Women and Registered Nurses in Aged Care

**DOI:** 10.1111/scs.70080

**Published:** 2025-07-03

**Authors:** Ariel Almevall, Karin Zingmark, Catharina Melander, Päivi Juuso

**Affiliations:** ^1^ Division of Nursing, Department of Health Sciences Mid Sweden University Sundsvall Sweden; ^2^ Division of Nursing and Medical Technology, Department of Health, Education and Technology Luleå University of Technology Luleå Sweden

**Keywords:** home care services, home environment, interpersonal relations, nursing care, psychological adaptation, qualitative research

## Abstract

**Aims and Objectives:**

With the expanding older population, the demand for home‐based aged care is increasing. While home care presents challenges, it also offers opportunities to transform aged care practices. This study, grounded in caring science and lifeworld philosophy, explores the meaning of the relationship between older women and registered nurses.

**Methods:**

Individual interviews were conducted with 11 women receiving home care, and a focus group was held with registered nurses working in the same setting. Data were analysed using qualitative content analysis.

**Results:**

The relationship between older women and registered nurses resembles a tapestry of emotional and physical connections, shaped by the women's vulnerability due to their dependence on care. Despite this, the women strove to be recognised as resilient and capable individuals.

**Conclusion:**

This study highlights the evolving relationship between older women and registered nurses in home care, shaped by the interplay of vulnerability and resilience. It offers insights into the relational dynamics in home‐based aged care and emphasises the importance of presence, attentiveness, and the recognition of the home as integral to the caring relationship.

## 1 Background

The nurse–patient relationship is essential in aged care. Aged care revolves around relationships and care approaches that enhance the value of the older person [1]. Research shows that relationships play a vital role in the health and well‐being of older adults, including those receiving home care [2, 3]. A lack of relationships may increase loneliness [4], which is marked by the subjective feeling of lacking companionship and is linked to confinement, future apprehension and depression [5]. Sya'diyah et al. [6] emphasise the role of registered nurses in alleviating loneliness, aligning with research on how relationships promote coherence and reduce loneliness [7, 8].

The alleviation of loneliness underscores the significance of the nurse–patient relationship in aged care and its impact on care quality. Strong relationships contribute to a sense of connectedness [9], built through recognising key qualities in the relationship [10]. These relationships in home care, characterised by unconditional support [11], have been shown to reduce hospital stays and improve satisfaction for both patients and registered nurses [12, 13].

Caring involves an understanding of the older person's experiences and a willingness to be present, fostering a sense of connectedness. Furthermore, connectedness is essential for the provision of compassionate care [14]. In the context of home health care, Blomqvist et al. [15] highlight compassionate care to involve ethical sensitivity leading to deeper meaning and improving competence in care. Devik et al. [16] found that compassion was essential in the effort of relieving suffering and promoting well‐being. In order to provide compassionate care, registered nurses have been shown to cultivate a deep connection with themselves, others, and the environment [17]. Living in one's own home has been associated with freedom, autonomy and preserved identity [18, 19, 20]. However, the relationship with care personnel can affect the older person's sense of at‐homeness and feelings of safety [21]. Single‐living older women with extensive nursing care possess valuable insights into the complexities of receiving aged care within a home‐based setting.

Aged care encompasses various aspects of daily life, including but not limited to personal hygiene, administration of medications, meal preparation, household upkeep and facilitation of social interactions alongside engagement in meaningful activities. The scope of care is vast. Aged care is created with the older person and their family and involves registered nurses, nursing assistants, care aides, physiotherapists, occupational therapists, physicians, social workers, among others. As the core of aged care, the relationship between registered nurses and single‐living older women with home care is a focal point for exploration. This study aims to explore the meaning of the relationship as described by older women and registered nurses.

## 2 Method

### 2.1 Study Design

The study employed a qualitative design, guided by the consolidated criteria for reporting qualitative research (COREQ) [22]. This design is grounded in caring science and lifeworld philosophy, both of which emphasise the holistic nature of patients as individuals whose experiences of health, suffering, and existential concerns are central [23]. Within this framework, values such as faith, hope and love are viewed as essential forces shaping care and creating an ethos of interconnectedness within the caring relationship [24].

The lifeworld approach informed our emphasis on understanding each participant's subjective experiences [25, 26]. Caring, in this view, is an ongoing dialogue with the lifeworld of the other, where meaning arises from lived experiences. This ensures that participants are seen in their wholeness, with full recognition of their relational and existential contexts.

The study also aligns with Eriksson's [27] principle that ethics precedes ontology. Ethical sensitivity was prioritised throughout the research process, with an clear commitment to safeguarding human dignity. Lévinas' [28] philosophy further guided this approach, positioning ethics as the foundation of understanding, where care is shaped by attentiveness to vulnerability and moral responsibility for the Other.

To complement these perspectives, the capabilities framework [29] informed the development of the interview guide. In particular, it guided the formulation of questions addressing agency, dignity and relational belonging in the context of the nurse–patient relationship. Anchored in a lifeworld perspective, we approached participants' experiences with openness and sensitivity, allowing meanings to emerge rather than being imposed.

The study design, sampling and data collection were grounded in a coherent philosophical and ethical framework. Reflexive dialogues within the research team strengthened interpretive rigour, and transparency was maintained through systematic documentation of analytical decisions and adherence to the COREQ checklist.

### 2.2 Participants and Recruitment

The study included older women in Sweden, aged 82–95 years (mean age = 90), all of whom lived independently and received home care. To meet the inclusion criteria, participants were required to receive a minimum of four home care visits per day, including weekends, from assistant nurses and care aides, as well as at least 15 h of care per week. Registered nurses visited between twice a week and twice a day, with additional visits provided as needed to address changes in care needs or routines. Women with cognitive impairments were excluded.

Recruitment was carried out in collaboration with coordinators who were familiar with the participants' circumstances, ensuring alignment with the inclusion criteria. As only women met these criteria within the given context, 12 were informed about the study and invited to participate. Those who expressed interest were referred to the researcher and received written information about the study before providing written informed consent. One woman declined, resulting in a final sample of 11 participants.

The sample size was guided by the concept of information power [30], ensuring that the data collected were sufficient to meet the aim of the study. All participants had previously cohabited and were receiving between 2.5 and 6 h of daily support, delivered through four to eight visits. The majority received 2.5–3 h per day, while only one participant received the full 6 h. Detailed participant characteristics are presented in Table 1.

**TABLE 1:** Participants' characteristics.

Age	Housing	Weekly care hours	Daily care hours	Daily home‐care visits
94	House	21	3	6
89	House	17	2.5	6
87	Apartment	15	2	4
82	Apartment	20	3	6
90	Apartment	22	3	6
89	Apartment	44	6	8
86	House	22	3	6
95	Apartment	17	2.5	6
90	Apartment	17	2.5	4
92	Apartment	22	3	6
94	Apartment	21	3	6

Registered nurses were purposively recruited from a municipal aged care organisation [31]. To be eligible, they were required to have a minimum of 2 years' experience in home care. Recruitment was facilitated by an administrator who was familiar with the staff and identified potential participants based on the inclusion criteria. The nurses received written information about the study and were invited to participate. Those who agreed provided written informed consent. In total, five registered nurses participated. They were between 35 and 47 years of age (mean age = 41) and had between 2 and 11 years of experience in home‐based care. Participant characteristics are presented in Table 1.

### 2.3 Data Collection

The study was grounded in a capabilities‐based approach [30], focusing on a good life for older women living at home with extensive support needs. In this study, the central theme was relationships with others. The primary question was: ‘Can you describe your relationships with others?’ Follow‐up questions, such as ‘Can you tell me more about that?’ and ‘Could you share an example?’ encouraged participants to elaborate and reflect more deeply.

Individual interviews were conducted with the older women in their own homes, a setting chosen to accommodate their limited mobility and ensure comfort [32]. The interviews were conducted side‐by‐side with the participants, overlooking their home environment or the view from their windows, fostering a familiar and calming atmosphere [33]. The interviews ranged from 21 to 61 min, with an average length of 38 min.

Data from registered nurses were collected through a focus group interview to foster dynamic discussion [34]. The group interaction encouraged participants to reflect on and expand upon each other's perspectives, thereby enriching the data on the studied phenomenon [34]. The focus group interview with the registered nurses lasted 92 min. They were asked: ‘How do you view the possibilities for older women to establish and maintain relationships with others?’ Follow‐up prompts, such as ‘Can you elaborate on that?’ and ‘Could you share an example from your professional experience?’ encouraged participants to expand on their reflections. All interviews were audio‐recorded and transcribed verbatim by the first author.

### 2.4 Data Analysis

A qualitative content analysis with a latent approach was used to explore deeper meanings in the data [35]. This method enabled the examination of nuanced aspects of participants' experiences, reflecting their existential realities while requiring a reflective stance from researchers. The analysis process involved several stages. Initially, the women's interviews were analysed by reading the transcripts repeatedly to identify recurring themes and patterns, capturing the essence of their interactions with registered nurses. This phase aimed to uncover both explicit and implicit meanings.

Next, the perspectives of the registered nurses were examined to understand how their experiences complemented or contrasted with those of the women. This involved coding the registered nurses' transcripts and identifying themes related to the caregiving relationship. The initial analysis of individual interviews and focus group data was conducted separately, allowing for the identification of themes unique to each dataset.

The synthesis was then conducted through iterative comparisons of the two datasets, allowing for the identification of patterns, contrasts, and complementary insights that captured the meaning of the relationships described by participants. This process enriched the analysis by enabling a deeper understanding through comparison between data and emerging themes. Regular discussions and critical reflections within the research team ensured interpretations grounded in the data and reflective of participants' meanings. This collaborative effort illustrated the interplay between researcher insights and the lived experiences captured in the study. To ensure rigour, the research team continuously returned to the data, engaged in interpretive dialogue, and maintained a reflective stance throughout the analysis. This process supported transparency and trustworthiness in the findings.

### 2.5 Ethical Considerations

The study was approved by the Regional Ethical Board in Umeå (No. LTU‐2706‐2015). Participants were provided with both written and verbal explanations of the study's objectives and procedures. They were informed that their participation was entirely voluntary and that they could withdraw from the study at any time without providing reasons. Participants were assured of the confidentiality of their information and were advised to contact the researcher with any concerns or questions.

The interviewer carefully monitored the participants' well‐being and responses during the interviews. If any signs of fatigue, disinterest or discomfort were observed, the participant was immediately asked if she preferred to skip a question, take a break or discontinue the interview and/or participation altogether. The health and well‐being of the participants were prioritised throughout the study, and no actions were taken that could potentially compromise these aspects.

## 3 Findings

The findings are presented through the overarching theme, ‘Nurturing Connection: Moving Beyond a Transactional Relationship’, followed by an exploration of the subthemes illustrated with interview quotes. Table 2 provides an overview of the themes and subthemes.

**TABLE 2:** Overview of the themes and subthemes.

Theme	Subtheme
Nurturing connection: Moving beyond a transactional relationship	The personal bond: Fostering presence and a comprehensive connection
	Caring for the home: Cultivating the relationship
	Beyond the threshold: Facilitating sense of place beyond home
	Balancing holistic health and disease‐oriented perspectives
	Unveiling vulnerability: Establishing a supportive connection

### 3.1 Nurturing Connection: Moving Beyond a Transactional Relationship

The relationship resembled a tapestry of connectedness. From the women's perspective, emotional closeness was essential and formed the foundation for meaningful relationships. In their reflections, they described the nurturing of emotional bonds and the comfort found in the presence of others. Registered nurses acknowledged the importance of physical closeness, highlighting its role in nurturing a sense of trust and enabling them to provide authentic care, differentiating it from a more transactional or purely procedural approach. They emphasised that such a level of closeness was essential for genuine, compassionate care, moving beyond a clinical or task‐focused dynamic.

Both groups underscored the importance of establishing genuine connections and creating a safe space for mutual understanding and growth. From the registered nurses' perspective, the challenges in the relationship were related to the evolving roles in caring for older women. This underscored the need to build timely relationships, communicate with empathy and presence, and embrace a more profound connection beyond the more transactional nature of care.

A tension emerged as women hesitated to burden registered nurses, who in turn acknowledged their own time constraints. This mutual concern reflected a shared desire to preserve the relationship amid practical challenges. However, a dichotomy emerged as women sought recognition for their competence and independence, while registered nurses predominantly acknowledged and emphasised their vulnerability. This divergence highlighted differing priorities: women aimed for acknowledgment of their capabilities, while registered nurses placed significance on understanding and tending to women's vulnerabilities in their care.

### 3.2 Subthemes

#### 3.2.1 The Personal Bond: Fostering Presence and a Comprehensive Connection

The experience of the women being marginalised by society and feeling alone either outside or within the community was prevalent in both perspectives. The women underscored the importance of their relationship with the registered nurse as a wellspring of meaningful interaction. They shared that their prior relationships had dissipated due to illness or bereavement, leaving them with scant social connections. The registered nurses, who visited the women frequently, sometimes multiple times a day and throughout the week, became consistent companions, fostering a profound bond.

We get to know each other quite well. We're there often—sometimes just occasionally, other times several times a day. In some cases, we've been visiting these women for more than 10 years. It's a unique experience. Registered nurse

The women's perspective revolved around their experiences of loneliness and the significance of the registered nurse's presence in their lives. On the other hand, the registered nurse's perspective encompassed the challenges of building and maintaining relationships with their patients amidst a high staff turnover. Within the relationship, there were descriptions of how both endeavoured to overcome hurdles that hindered them from developing or sustaining a connection. Part of this challenge lay in the difficulty of forging a deeper connection due to the feelings of stress, loneliness, and isolation that arose from the time constraints surrounding their interactions. However, it was not solely about difficulties in establishing a connection; the circumstances also exacerbated the women's sense of loneliness, making it feel even more profound as they endured rushed visits in their already solitary existence. The women described how they attempted to limit their demands and needs, so as not to rush the nascent stages of their relationship with the registered nurses.

*Woman*: I'd like to have more time to sit and talk—about everything. But you can't really do that … It's more like occasional chats here and there—short ones.

*Interviewer*: Would you like that?

*Woman*: Sure, but you can not really ask for it.

*Interviewer*: Why not?

*Woman*: Well, I can ask … one can ask.

*Interviewer*: Is there a risk in asking for something you might not get?

*Woman*: That you might be disappointed … yeah.

*Interviewer*: Is it hard to risk being disappointed?

*Woman*: Yeah, it is better to just keep quiet…

Conversely, the registered nurse described how she tried to prioritise those who were the most seriously ill, delaying the initiation of relationships with patients who were somewhat healthier. However, both strategies came at a cost. The women adjusted their daily lives based on the registered nurse's availability, while the registered nurse felt that the relationships with the patients did not have the opportunity to commence early enough, wishing that the relationship could begin before the care needs became extensive.

Further, both described emotional nurturing, empathetic communication and physical presence, underscoring the importance of emotional support and its impact within the relationship between the older woman and the registered nurse. For older women, registered nurses served as pillars of emotional support by offering understanding and empathy, a safe space to express their feelings and seek solace. This nurturing environment allowed older women to feel valued, heard, and supported and fostering a sense of comfort. The women appreciated the registered nurses' genuine interest and compassionate communication, which often helped them to reflect on their experiences and find closure where needed. Moreover, the physical presence of registered nurses holds significant importance for older women. Whether through gentle touch or simply being present, registered nurses' physicality reassures and reinforces a sense of connection, emphasising the nurturing aspect of the relationship.

The theme showcases registered nurses as pillars of emotional support, offering a safe space for expression and solace, fostering profound comfort and value for older women. Conversely, registered nurses emphasised the importance of comprehensively understanding the woman's nuances, including movement patterns and unspoken cues, to establish a more impactful relationship centred on meeting her needs for support and care.

#### 3.2.2 Caring for the Home: Cultivating the Relationship

The home environment was evident in both perspectives. The women described feeling cared for and valued when registered nurses attended to their homes. The registered nurses also expressed how they gained insights into the women's lives by engaging with their home environment and immediate surroundings. By observing and interacting within the women's home setting, the registered nurses described that they developed a deeper understanding of the women's lifestyle, interests, and needs. The home and immediate surroundings provided the registered nurses with additional insights into the relationship. The home was described as an extension of both the woman's identity and an expression of the relationship with the registered nurses.

… but some registered nurses don't notice or take the initiative. I probably don't sound very angry, but they can tell I'm irritated. For example, if I haven't been in the kitchen, I don't know how it looks. So I can't tell how it's been for several days. Today, when someone was here, I said: “If there's anything lying around in the kitchen or the fridge, just throw it out.” I feel embarrassed about how things might look at home when I don't even know myself what the kitchen looks like. Woman

Simultaneously, the registered nurses emphasised the importance of paying attention to and caring for the women's homes as a way to build and strengthen their relationship with the women. They highlighted how attending to the home environment, such as recognising and appreciating small changes the women made, like decorations for holidays or seasonal transitions, was integral to fostering a deeper connection. The registered nurses also described how they assisted with minor tasks around the home, such as changing lightbulbs or addressing other small maintenance needs. These actions were not only aimed at ensuring the women's comfort but also at reinforcing the bond between the registered nurses and the women by demonstrating attentiveness and personal care.

I think a home says a lot about who we are. It's important to show that we care by noticing the small things she's done—or the little issues that might need fixing. That helps us understand her better and provide more thoughtful care. Registered nurse

In caring for the home, both the registered nurse and the woman cultivated more than physical space; they cultivated trust, respect and reciprocity. This sanctuary became an emblem of their shared relationship, a space where care for the home echoed care for the woman herself. Tending to the home became a gesture of reverence and care for each other.

#### 3.2.3 Beyond the Threshold: Facilitating Sense of Place Beyond Home

In the relationship between older women and registered nurses, the depth of their bond created an unforeseen conduit, allowing the women to transcend the boundaries of their homes. These women, constrained by physical limitations, found newfound avenues to explore beyond their homes due to the nurturing bond forged with their registered nurses.

This relationship not only facilitated practical opportunities for these women, who otherwise could not venture out independently. It provided the women with an alternate odyssey. Through conversations encompassing everyday life, reminiscences, and the bustling cityscape, it felt as though they could traverse realms without physically departing their home.

Sharing what you've seen on the way to her home—like the mall being renovated or something happening in the world—helps take the conversation beyond the home. It makes the relationship feel more personal … it's not just about care. Registered nurse

For these women, it transcended mere acquaintanceship with life beyond their thresholds; it encapsulated sharing personal anecdotes and cherished memories from days gone by. These exchanges and shared anecdotes evoked a sense of being present in spaces beyond the confines of their own rooms. For the registered nurse, it wasn't just about recounting personal anecdotes but also about imparting a glimpse of the external world to these women. This connection transcended the physical confines of home, reshaping their conversations and emotions. It served as a gateway for these women to engage with the world beyond their homes.

#### 3.2.4 Balancing Holistic Health and Disease‐Oriented Perspectives

Relationships were described as providing women with a sense of wholeness and well‐being, where they felt more than just a patient in need of healthcare. Support from the registered nurse encouraged a shift in the healthcare approach, demanding a focus beyond just treating the illness and instead considering the overall well‐being and wholeness of the patient.

*Interviewe*r: It sounds like it is challenging to make decisions, because you need to contact many people, figure out what support you want, and get the things you need.

*Woman*: I am always trying. And the registered nurse—do you know her? She used to be my nurse.

*Interviewer*: I have met her.

*Woman*: Oh, I love her, I really do … I can't believe she has to leave. I've never met anyone like her before … never. And she felt the same way. She actually called me the other day, and she was … just wonderful. Everyone says that. You know, the chemistry has to be right … I don't know what she has, but wow. I was almost in tears.

Well—let us continue.

*Interviewer*: That question about decision‐making—I interpret your story as feeling very restricted and unable to make decisions.

*Woman*: Exactly. Because I want to decide how I want things to be. I mean, I want to live … can you imagine living like this? And then the registered nurse said to me afterwards, “It's good that you stood your ground.” Don't you understand—when I say no, I mean it. I want to live at home and have the support I need. Woman

The registered nurse's commitment and interest in the women's lives contributed to making them feel more than just a body, or a number in the healthcare system. However, despite this, the registered nurses noted an increased emphasis on managing illness and addressing risks, which sometimes led to a more fragmented approach to care. This focus on specific health issues occasionally affected the overall cohesion of the women's care.

We have to check so much, measure everything, and take things away if they make even the smallest mistake or forget something. We focus so much on the illness—why not also look at the healthy side? Let them try, even if they make mistakes, and remind them instead of taking over. They're living their lives at home, not in a hospital. I don't think that's right… Registered nurse

The women described how their relationship with the registered nurses provided them with an opportunity to reflect on their lives. They experienced reciprocity in the relationship when registered nurses showed genuine interest in their stories. Through this relationship, the women found meaning and understanding in events and experiences throughout their lives.

#### 3.2.5 Unveiling Vulnerability: Establishing a Supportive Connection

The dynamics of vulnerability and the establishment of a supportive bond within the relationship hint at how women perceived the respect and appreciation from registered nurses. Women often regarded registered nurses as trusted confidants, with whom they could openly share their concerns and desires.

I've known her for years … she knows more about me than my husband ever did (giggles). We've sort of become friends—it just happened that way. Maybe it's because she understands what us older folks are like. But it really feels like she genuinely cares about me. We can talk about everything under the sun. Woman

This mutual understanding and sense of companionship fostered a deeper, more meaningful connection. However, descriptions of power dynamics and emotional suppression where registered nurses unintentionally conveyed reprimands or inadvertently silenced women were described to lead to feelings of sadness and a perceived loss of agency.

The evolving nature of the relationship was described as a continuous transition, moving from revealing vulnerability to building a supportive and trusting bond. The women described their appreciation when registered nurses acknowledged and occasionally recognised the journey they were on, how far they have come, and that they are still progressing. Simultaneously, they expressed concerns about how the journey will unfold and how their relationship with registered nurses will evolve.

It's really important that we get to know each other. When a new registered nurse comes in, it's crucial to build a relationship as soon as possible. Registered nurse

In the relationship, older women often depended on registered nurses for assistance with daily tasks, outings and social interaction. While this reliance brought both joy and anxiety, the women expressed a desire for greater control over their daily lives and for nurses who actively supported their individual needs and preferences. Uncertainty and stress arose when familiarity between registered nurses and older women was lacking due to frequent personnel changes. To alleviate this uncertainty, registered nurses prioritised continuity of care and sought to establish a consistent presence. In doing so, they aimed to build trust and reduce stress for the women, thereby ensuring a sense of stability and familiarity in their care.

## 4 Discussion

This study explored the meaning of the relationship between older women and registered nurses within home‐based aged care. The findings demonstrate that these relationships extend beyond routine care tasks; they are emotional, existential, and relational in nature, shaped by an ongoing negotiation between dependence and agency, vulnerability and resilience. Situated in the intimate and personal space of the home, the nurse–patient relationship becomes a site of connection, recognition and meaning‐making.

These findings resonate with earlier research highlighting the significance of relational care in aged care settings. Santini et al. [36] show how the quality of social relationships is closely linked to mental health and well‐being in later life, reinforcing the need for meaningful interpersonal connections. Sundström et al. [37] emphasise that trust and mutual understanding are essential to building strong nurse–patient relationships in home care, identifying these bonds as central to the quality of care delivered. Similarly, Mathiesen et al. [38] illustrate how registered nurses' relationships with older adults often evolve beyond conventional caregiver roles, becoming comprehensive and empathetic interactions grounded in presence, emotional attunement and ethical sensitivity. The centrality of the registered nurse–patient relationship reinforces its meaning in home care [16, 39, 40]. McGarry [41] highlights the nuances involved in establishing meaningful connection, such as in defining roles, relationships and boundaries between older people and registered nurses within home care. Moreover, studies such as those by Iwasaki et al. [42] and Jones and Moyle [43] underscore the dynamics of the relationship within aged care settings.

The study's findings align with prior research on loneliness [6, 44]. Various factors, including the healthcare personnel's proficiency in establishing meaningful relationships [5], can be linked to feelings of loneliness among older persons with home care. To alleviate the negative repercussions of loneliness, registered nurses could cultivate an understanding of older persons' needs and create an environment conducive to fostering a sense of connection.

The findings in this study emphasise the emotional closeness between older women and registered nurses. The findings centre around the dichotomy between acknowledging competence and emphasising vulnerability, revealing how both women and registered nurses navigate the complexities of these roles. This exploration underlines the importance of moving beyond transactional relationships to foster a genuine, compassionate connection. From a lifeworld perspective, Galvin [23] underlines the importance of understanding the existential experiences of well‐being and suffering, emphasising the need for a vocabulary that captures these experiences, focusing on how caring practices can be attuned to the deep, often overlooked aspects of human life, such as dignity, vulnerability and freedom.

While the findings align with prior research, this study provides insights by highlighting the dual focus on competence and vulnerability within the registered nurse–patient relationship. Specifically, the results illustrate how these dynamics are not only negotiated within care interactions but also shape the relational and emotional outcomes for both older women and registered nurses. Furthermore, the role of the home environment as an extension of relational identity, rarely emphasised in existing literature, emerges as a significant contributor to fostering trust and connection in home‐based aged care. While previous research has acknowledged the importance of “feeling at home” or remaining at home in old age (e.g., [19, 21]), this study reveals a more dynamic and relational dimension: the home emerges not only as a backdrop for care, but as an expressive, co‐constituted space within the registered nurse–patient relationship. In attending to the home, registered nurses engage not just with material surroundings, but with the woman's lived identity, memory and agency. This attentiveness becomes a moral and relational act, one that invites the older woman to participate in care not only as a recipient, but as a host, a person with presence and place. Thus, the home is not merely a site of care, but a site for mutual recognition and becoming, deepening the ethical fabric of the relationship. This expands current understandings of relational care in home settings and opens up pathways for developing existentially grounded caring practices.

The findings in this study highlight how registered nurses acknowledge the significance of physical closeness and emotional engagement in building trust and providing authentic care. Galvin [23] argues for an integrated approach to knowledge, where empathy (the heart), understanding (the head) and actionable steps (the hand) are all essential in caring practices. Advocating for empathic imagination, caregivers are encouraged to deeply engage with the experiences of those they care for, thus promoting a holistic approach to care.

This study further demonstrates a tension in the relationship as older women are reluctant to burden registered nurses, who are also constrained by time limitations. This mutual concern reflects the challenges in maintaining the quality of care amid practical constraints, which shows a need for balancing the acknowledgment of patients' competence and their vulnerabilities, highlighting a nuanced understanding of patient autonomy and support. This tension between autonomy and vulnerability also reflects key elements of the capabilities approach [30], where agency, dignity and relational belonging are seen as central to human flourishing, particularly in contexts of care. Galvin [23] emphasises the need for existential knowledge in care, which involves understanding the deeper aspects of patients' experiences beyond mere clinical symptoms.

The findings in this study and Galvin's perspectives highlight different facets of the caring relationship. The findings in this study focus on the practical and emotional dynamics between registered nurses and older women, emphasising the importance of moving beyond a transactional relationship. In contrast, Galvin provides a broader philosophical framework, advocating for an existential approach to care that includes a deeper, more nuanced understanding of human experiences. Together, these perspectives suggest a comprehensive approach to caregiving that values emotional connection, empathic understanding, and a deep respect for the existential aspects of the human condition.

## 5 Method Discussion

The strength of this study lies in the participants' substantial firsthand experiences—either as older women receiving extensive home care or as registered nurses providing it. These perspectives offer depth and insight into caregiving relationships.

To enhance trustworthiness, this study adhered to key principles of credibility, dependability and transferability [35]. Credibility was ensured through purposeful selection of participants, in which older women with extensive home care needs and experienced registered nurses were included. Additionally, open‐ended questions allowed participants to describe their experiences in their own words, reducing the risk of researcher‐imposed interpretations. Furthermore, care was taken to define meaning units at an appropriate level of abstraction to avoid losing depth through excessive fragmentation while maintaining clarity in coding and analysis.

Dependability was maintained through a structured and consistent data collection process. Interviews with the older women were conducted in their homes to promote a comfortable and natural setting, while the focus group with registered nurses fostered reflective discussions. To ensure analytical consistency, an iterative approach was applied, involving repeated readings of transcripts, independent coding by multiple researchers, and regular discussions within the research team to refine emerging themes. All analytical decisions were documented to maintain transparency and ensure consistency throughout the analysis process.

Transferability was supported by detailed descriptions of the study context, including participant characteristics, the home care setting, and the broader aged care framework. Thick descriptions allow readers to assess the applicability of the findings to other contexts. In addition, direct participant quotations were included to illustrate key findings, providing readers with insight into the lived experiences of the participants and further enhancing transferability.

The sample size was guided by the concept of information power [30], depended on factors such as the specificity of the study aim, the richness of participant experiences, and the quality of dialogue. In this study, information power was enhanced by clear inclusion criteria, ensuring that participants had extensive and relevant experience of home care relationships. However, a potential limitation is that all participants were women, which may narrow the applicability of findings to broader home care settings where male recipients are also present. Additionally, while the study provided rich narratives, the lack of follow‐up interviews may have limited opportunities to refine emerging insights during data collection.

## 6 Conclusion

This study reveals how registered nurse–patient relationships in home‐based aged care go beyond routine transactions and are shaped by older women's vulnerability, resilience, and the home as an extension of their identities. Acknowledging both competence and vulnerability fosters trust and emotional closeness, enhancing well‐being for both older women and registered nurses. These findings underscore the importance of a relational approach that integrates existential dimensions, ensuring compassionate care.

This study illustrates how the home, far from being a passive backdrop, becomes a living and expressive element in the nurse–patient relationship, a site through which dignity is affirmed, trust is co‐created, and care unfolds as a mutual, situated practice. For the woman, the home is not only where life continues, but where she actively expresses who she is. When the registered nurse meets this expression with attentiveness, not as a task to complete, but as an invitation to enter, the relationship deepens. In this light, care is not complete without entering into the shared space of the home, not to assess it, but to be with it—as part of being with her. Without this, the woman risks being reduced to her needs, and the nurse to her duties. But when the home is embraced as a relational field, it becomes not just the place where care happens; it becomes part of what care means.

In light of these findings, several practical implications emerge. First, fostering relational care requires a balanced recognition of both the competence and vulnerability of older women in home‐based settings. Organisations can support this by equipping registered nurses with the tools and time to cultivate meaningful connections. Second, continuity of care should be prioritised to build trust and reduce uncertainty for older women, particularly in the face of staff turnover. Finally, the home is not just a physical space but an extension of the older woman's identity and a vital aspect of the caring relationship.

While this study focused on older women, future research may offer further insight into how care relationships are experienced by older men living at home. Further research could also examine how care organisations might support the cultivation of existentially grounded relationships, and how such long‐term connections affect the emotional and ethical well‐being of registered nurses. In addition, examining the home as a co‐created relational space may offer deeper insight into how care is not merely delivered, but lived, and how belonging is shaped and reshaped through the rhythms of everyday life.

## Author Contributions

A.A., P.J. and K.Z. contributed to the study design. A.A. collected the data. A.A. conducted the analysis together with K.Z., C.M. and P.J. A.A. and P.J. regularly met to discuss all steps in the analysis process. All authors discussed and revised the draft of the manuscript.

## Ethics Statement

The study was approved by the Regional Ethical Board in Umeå on October 12, 2015 (No. LTU‐2706‐2015).

## Conflicts of Interest

The authors declare no conflicts of interest.

## Data Availability

Research data are not shared.
